# Editorial: Adverse and toxic effects of childhood cancer treatments, volume II

**DOI:** 10.3389/fonc.2025.1597172

**Published:** 2025-04-11

**Authors:** Antonio Ruggiero, Roderick Skinner, Pinki Prasad

**Affiliations:** ^1^ Pediatric Oncology Unit, Fondazione Policlinico Universitario Agostino Gemelli IRCCS, Università Cattolica del Sacro Cuore, Rome, Italy; ^2^ Department of Paediatric and Adolescent Haematology/Oncology, Great North Children’s Hospital, Newcastle, United Kingdom; ^3^ Newcastle University Centre for Cancer, University of Newcastle, Newcastle, United Kingdom; ^4^ Department of Pediatrics, Division of Pediatric Hematology/Oncology, Louisiana State University (LSU) Health Sciences Center New Orleans, Manning Family Children’s, New Orleans, LA, United States

**Keywords:** late effects, children, cancer, treatment, survivors (long-term survivors)

The growing number of childhood cancer survivors has unveiled both acute and delayed toxicities specific to various treatment modalities, significantly impacting their quality of life. As cancer therapies become more effective and patient life expectancies rise, achieving a balance between oncologic efficacy and the reduction of acute and chronic toxicities has emerged as a crucial priority (1, 2). Approximately 50 years ago, in their pivotal 1974 publication, Meadows and D’Angio outlined the extensive range of potential adverse long-term effects of successful treatment for childhood cancer (3). Over the past two decades, numerous research studies have greatly improved our understanding of the acute and long-term impacts of childhood cancer and its treatment (4–8).

This Research Topic focuses on emerging research and clinical strategies aimed at improving our understanding of the adverse effects of childhood cancer treatments. The Research Topic includes original investigations and review articles contributed by leading experts, offering both preliminary findings and promising approaches to mitigate toxicities. The studies presented cover a broad range of issues, from identifying risk factors for toxicity to exploring novel therapeutic strategies that minimize harm while maintaining the effectiveness of cancer treatments.

A key area of this research is the exploration of commonly used chemotherapeutic agents in pediatric oncology and their associated toxicities. Methotrexate (MTX), a fundamental treatment for childhood cancers like acute lymphoblastic leukemia (ALL) and osteosarcoma, is highly effective but also known to cause severe side effects. High-dose MTX (greater than 500 mg/m²) can lead to nephrotoxicity, with renal function and fluid balance playing a critical role in its elimination. Research by Mosleh et al. has shown that genetic polymorphisms, such as those in the SLC19A1, MTHFR, and SLCO1B1 genes, can influence MTX metabolism. These findings suggest that genetic profiling, combined with careful monitoring of kidney function and hydration, may help optimize MTX therapy, reducing adverse effects and improving patient outcomes.

Mercaptopurine, another important chemotherapeutic drug used to treat leukemia, is metabolized differently depending on genetic factors. Muhammad et al. examined the impact of the NUDT15 (c.415C>T) genetic variation on mercaptopurine metabolism, which affects both the necessary dosage and the efficacy of treatment. This highlights the value of personalized medicine in pediatric oncology. By tailoring treatment to individual genetic profiles, toxicity can be minimized, and therapeutic effectiveness can be maximized, offering a more targeted approach to cancer care.

Cisplatin, a potent chemotherapy agent used in the treatment of pediatric solid tumors, poses significant challenges due to its potential to cause ototoxicity and nephrotoxicity. Meijer et al. have investigated the use of sodium thiosulfate (STS) as an otoprotectant to reduce hearing loss associated with cisplatin. Their research emphasizes the importance of precise dosing, timing, and formulation to ensure that STS effectively protects against hearing loss without interfering with cisplatin’s anticancer effects. Ongoing clinical trials will be crucial in refining these protocols to ensure that the benefits of cisplatin are not overshadowed by its toxicities.

For childhood cancer survivors, long-term cognitive impairments are an ongoing concern, especially among those treated with platinum-based agents such as cisplatin, anti-metabolite chemotherapy, or exposed to CNS radiation. Research by L’Hotta et al. found that even survivors who did not receive cranial radiation or suffer from central nervous system (CNS) tumors experienced significant cognitive deficits. These findings underscore the importance of broader surveillance of cognitive health in childhood cancer survivors and highlight the need for early interventions to manage these issues and improve quality of life.

Ototoxicity, especially hearing loss, continues to be a major concern for childhood cancer survivors, significantly affecting their daily lives. Spence et al. compared two assessment tools—HEAR-QL and PROMIS—and found that HEAR-QL was more effective at detecting impairments in quality of life due to hearing loss. This reinforces the importance of incorporating quality of life assessments into survivor care, particularly for those at risk of auditory impairments, to better manage and improve their long-term health outcomes.

Supportive care during and after cancer treatment is another increasingly important area of focus. Katabalo et al. conducted a study in Tanzania, which found that medications such as ondansetron and saline were commonly prescribed to manage chemotherapy side effects. However, the study revealed that national cancer treatment guidelines lacked comprehensive recommendations for supportive care, highlighting the need for more detailed and standardized protocols. Developing these guidelines will ensure that chemotherapy side effects are managed more effectively across the country, improving the overall experience and outcomes for patients.

As immunotherapies like Tisagenlecleucel gain traction in treating relapsed or refractory B-cell ALL, new toxicities are emerging. Wang et al. examined the cardiovascular adverse events (CVAEs) associated with this treatment and found that these events can occur rapidly, especially in patients on concomitant medications. Their study emphasizes the importance of vigilant monitoring for cardiovascular toxicity, particularly in the early stages of treatment, to mitigate risks and enhance patient safety. Longitudinal studies monitoring patients who receive these therapies is important to capture long term adverse effects.

Fertility preservation is becoming an increasingly important consideration for adolescent cancer patients. Rodriguez-Wallberg et al. highlighted the gender-specific challenges of fertility preservation procedures, particularly for female patients who may face discomfort from ovarian stimulation and distress from gynecological interventions. Their study stresses the importance of providing tailored communication and support to reduce fertility-related distress, improving the overall experience for adolescents undergoing fertility preservation.

As this Research Topic concludes, we would like to express our sincere gratitude to all the authors, researchers, and reviewers who contributed to this collection. Each article has significantly enriched our understanding of the adverse effects of childhood cancer treatments and provided valuable insights into improving the long-term care of survivors. The dedication and expertise of these contributors are essential in shaping the future of pediatric oncology care.

In conclusion, while advancements in cancer treatment have drastically improved survival rates for children, these treatments often come with long-term health challenges. This body of research serves as a call to action, emphasizing the need for ongoing innovation in both therapeutic or preventive strategies and supportive care to minimize the long-term toxicities of childhood cancer treatments. By focusing on personalized medicine, early intervention, and comprehensive care, we can help ensure that childhood cancer survivors not only live longer but also lead healthier, more fulfilling lives.
